# The evolving process of ferroptosis in thyroid cancer: Novel mechanisms and opportunities

**DOI:** 10.1111/jcmm.18587

**Published:** 2024-08-20

**Authors:** Lin Yin, Xiaodan Luo, Xian Zhang, Bomin Cheng

**Affiliations:** ^1^ Thyroid Gland Breast Surgery Shenzhen Traditional Chinese Medicine Hospital Shenzhen China; ^2^ Department of Hemodialysis Huangshi Central Hospital Huangshi China; ^3^ Department of Neurology, Affiliated Zhongda Hospital Research Institution of Neuropsychiatry, School of Medicine, Southeast University Nanjing Jiangsu China; ^4^ Chinese Medicine Health Management Center Shenzhen Traditional Chinese Medicine Hospital Shenzhen China

**Keywords:** ferroptosis, ferroptosis‐related gene, ferroptosis‐targeted therapy, lipid peroxidation, thyroid cancer

## Abstract

Thyroid cancer (TC) is a prevalent endocrine malignancy, with a significant increase in incidence worldwide. Ferroptosis is a novel form of programmed cell death, primarily caused by iron overload and reactive oxygen species (ROS)‐dependent accumulation of lipid peroxides. The main manifestations of cellular ferroptosis are rupture of the outer membrane, crumpling of the mitochondria and shrinkage or disappearance of the mitochondrial cristae, thus leading to cell death. Ferroptosis is an important phenomenon in tumour progression, with crosstalk with tumour‐associated signalling pathways profoundly affecting tumour progression, immune effects and treatment outcomes. The functions and mechanisms of ferroptosis in TC have also attracted increasing attention, mainly in terms of influencing tumour proliferation, invasion, migration, immune response, therapeutic susceptibility and genetic susceptibility. However, at present, the tumour biology of the morphological, biological and mechanism pathways of ferroptosis is much less deep in TC than in other malignancies. Hence, in this review, we highlighted the emerging role of ferroptosis in TC progression, including the novel mechanisms and potential opportunities for diagnosis and treatment, as well as discussed the limitations and prospects. Ferroptosis‐based diagnostic and therapeutic strategies can potentially provide complementary management of TCs.

## 1 BACKGROUND

**FIGURE 1:**
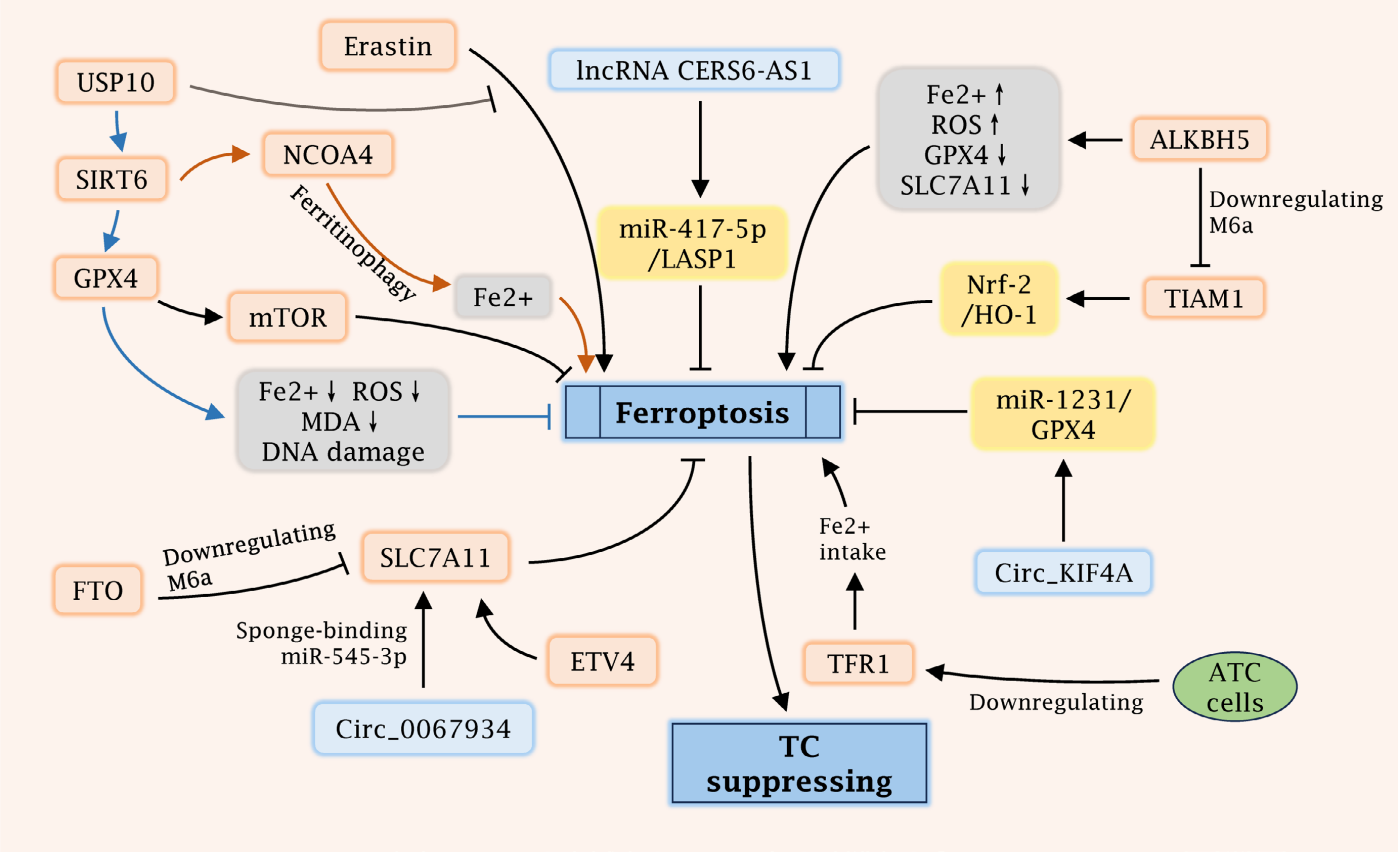
Classification and immune microenvironment of thyroid carcinoma. Based on the origins and extent of differentiation, thyroid cancer could be classified as papillary thyroid carcinoma (PTC), follicular thyroid carcinoma (FTC), medullary thyroid carcinoma (MTC) and anaplastic thyroid cancer (ATC). The immune microenvironment in thyroid carcinoma is a complex and dynamic system, where host cells coexist with tumour cells and act through mutual interactions to promote or inhibit tumour progression. This interaction involves the secretion of various cytokines, chemokines and antibodies from B cells. Additionally, immune cells could also directly contact tumour cells to exert anti‐tumour effects.

Thyroid cancer (TC) is the most common endocrine cancer, with a significant increase in incidence worldwide.^1^, ^2^ Based on differences in tumour origin and differentiation, TCs are subdivided into papillary thyroid carcinoma (PTC), follicular thyroid carcinoma (FTC), medullary thyroid carcinoma (MTC) and anaplastic thyroid cancer (ATC).^3^, ^4^ Of these, PTC is the most highly prevalent type of TC with a favourable prognosis, whereas ATC is less prevalent but is the most aggressive and lethal type of TC.^5^, ^6^ The different types of TC are highly heterogeneous between and within tumours, as evidenced by histopathological differences and genetic as well as epigenetic alterations.^7^, ^8^ Multiple factors, including thyroid hormones, Iodine intake and genetic factors, play a pivotal role in the pathogenesis and progression of TC, influencing cellular proliferation, differentiation and metabolic regulation within the tumour microenvironment.^9^, ^10^, ^11^, ^12^ Additionally, the immune microenvironment plays a crucial role in the progression and metastasis of TC.^13^ Multiple immune cells, including macrophages, natural killer (NK) cells, mast cells, various subtypes of T cells and dendritic cells (DCs), interact with TC cells and stromal components collectively, consequently influencing tumour growth and response to therapy.^14^, ^15^ The presence of tumour‐infiltrating lymphocytes (TILs) and the expression of immune checkpoint molecules such as PD‐1 and CTLA‐4, as well as those key secretory factors, are indicative of the immune landscape within TC tumours.^16^ These interactions can modulate the tumour microenvironment, affecting processes such as angiogenesis, immune evasion and metastatic potential.^17^, ^18^ Understanding the dynamics of the immune microenvironment is essential for developing effective immunotherapeutic strategies for TC. The development of risk stratification systems for thyroid nodules as well as new treatment options for TC has been in full swing in the past to provide the basis for evidence‐based clinical practice guidelines for TC.^19^ However, basic TC‐based biological research remains an important cornerstone for the development of new approaches to the early diagnosis and treatment of TC (Figure 1).

Ferroptosis is a novel, highly publicised, modality that regulates cell death, primarily caused by iron overload and reactive oxygen species (ROS)‐dependent accumulation of lipid peroxides.^20^, ^21^ The common cellular features of ferroptosis are smaller mitochondria, reduced mitochondrial cristae, increased mitochondrial membrane density and increased rupture of mitochondrial membranes.^22^ Ferroptosis can be initiated by two pathways, an exogenous transporter protein‐dependent pathway and an endogenous enzyme‐regulated pathway.^23^, ^24^ The phenomenon of ferroptosis is widespread in humans, mammals, plants and fungi and humans are involved in the progression of virtually all diseases including neurological disorders, infectious diseases and tumours.^25^, ^26^ Ferroptosis is involved in the progression of almost all tumours, including breast, lung, liver, ovarian and TCs.^27^, ^28^, ^29^ Comparatively speaking, ferroptosis has been well studied in high‐incidence and malignant tumour types and less well studied in low‐incidence and benign tumours.

Ferroptosis is potentially a novel cancer therapeutic target that augments tumour cell death and overcomes drug resistance through synergistic effects with other programmed cell death (PCD) pathways.^30^, ^31^ The roles and mechanisms of ferroptosis in tumour suppression and tumour immunity have grown in prominence, and therapeutic strategies targeting tumour ferroptosis have been developed and explored based on the susceptibility of tumour cells to ferroptosis.^32^ The function and mechanism of ferroptosis in TC are also gaining attention. Genetic polymorphisms in genes associated with ferroptosis are associated with TC susceptibility. For example, APOE‐rs429358, BCL3‐s34698726 and rs8100239 have been implicated in elevated TC risk.^33^ In different types of TC, ferroptosis‐associated markers differ in abundance and are intimately tied to the characteristics of the disease. Sun et al.^34^ identified 204 differentially expressed proteins (DEPs) between follicular adenoma (FA) and FTC by proteomics, of which 31 proteins were effective in distinguishing follicular tumours. Pathway enrichment analysis for 204 DEP showed that ferroptosis was the most enriched and activated pathway for these proteins.

At present, the current morphological, biological and mechanistic pathways of ferroptosis are increasingly arising in tumour biology.^35^, ^36^ However, the multiple mechanisms, fine‐tuning and immunoregulation involved in the ferroptosis process have not yet been summarised in sufficient depth in TC. Therefore, we highlighted the evolution of ferroptosis in TC, including the novel mechanisms and potential opportunities for diagnosis and treatment, as well as discussed the limitations and prospects. Ferroptosis‐targeted therapeutic strategies may shed new light on the treatment of malignant TC.

## 2 AN OVERVIEW OF IRON AND FERROPTOSIS

Ferroptosis is a new form of cell death first defined in 2012 and differs from traditional cell death modes such as apoptosis and autophagy.^37^ The most prominent features of ferroptosis are intracellular iron‐overloading and accumulation of lipid peroxides (LPO). Iron‐overloading is a key trigger of ferroptosis, and the accumulation of LPO could result in damaged plasma membranes.^38^ Morphologically, ferroptosis does not exhibit the typical morphological features of apoptotic cells, such as chromatin breaks.^39^, ^40^ Rupture of the outer membrane, crumpling of the mitochondria and reduction or disappearance of the mitochondrial cristae are key characteristic changes that occur in ferroptosis.^41^, ^42^ Recent studies have demonstrated that the activation of selected autophagy is involved in the core mechanism of ferroptosis, suggesting some kind of cross‐over and regulatory relationship between iron apoptosis and other forms of PCD.^37^, ^38^

Ferroptosis is inherently the result of cellular damage caused by dysregulation of the intracellular oxidative and antioxidant balance.^43^ Iron is a constituent of many intracellular metalloproteins and participates in processes such as oxygen transportation, ATP production, DNA synthesis and repair and substance metabolism.^44^ However, excessive intracellular iron could generate ROS via the Fenton reaction, leading to cell damage.^45^ Therefore, maintaining normal iron homeostasis is indispensable for cell survival.

Extracellular iron could bind to transferrin in the form of trivalent iron ions, which are then recognised and bound by transferrin receptors located on the cell membrane.^46^ These complexes develop endosomes, which in turn are transported to the lysosome. In the acidic environment within the lysosome, trivalent iron ions dissociate from transferrin and subsequently get reduced to divalent iron ions by the six transmembrane epithelial antigen of prostate family member 3 (STEAP3).^47^ Through the divalent metal transporter protein 1 (DMT1), divalent iron ions travel across the lysosomal membrane into the cytoplasm, where these highly chemically reactive and redox‐active iron ions form a labile iron pool (LIP).^48^ Extracellular divalent iron could be directly entered into the cytoplasm through the transmembrane DMT1; furthermore, cytoplasmic haemoglobin could be degraded to release divalent iron as well, jointly participating in the composition of the LIP.^49^ Iron in LIP could be transported out of the cell via ferroportin, a membrane iron transport protein located on the cell membrane, or be stored by binding to ferritin, thereby maintaining the balance of cellular iron ions.^50^, ^51^ Ferritin could in turn undergo NCOA4‐mediated degradation, which increases the amount of unstable intracellular iron.^52^ When LIP levels exceed the iron homeostatic range, abundant unstable irons generate ROS via the Fenton reaction. In the presence of endogenous ROS generated by other metabolic processes, these ROS can lead to lipid peroxidation of phospholipids containing polyunsaturated fatty acids (PUFA), thereby initiating ferroptosis.^53^

Lipid peroxidation is the core process of ferroptosis, in which PUFAs serve as the main substrate engaged in cellular lipid peroxidation.^54^ Acyl‐CoA synthetase long‐chain family member 4 (ACSL4) and Lysophosphatidylcholine Acyltransferase 3 (LPCAT3) catalyse free arachidonic acid (AA) and adrenic acid (AdA) to form AA/AdA‐membrane phosphatidylethanolamine (PE), which subsequently undergoes lipid peroxidation via either enzymatically or non‐enzymatically.^55^, ^56^, ^57^, ^58^ Enzymatic processes are primarily mediated by arachidonic acid lipoxygenases (ALOXs), while non‐enzymatic processes are dominated by ROS.^59^ Together, these reactions enable the accumulation of LPO and the damage to the plasma membrane, ultimately causing ferroptosis.

**FIGURE 2:**
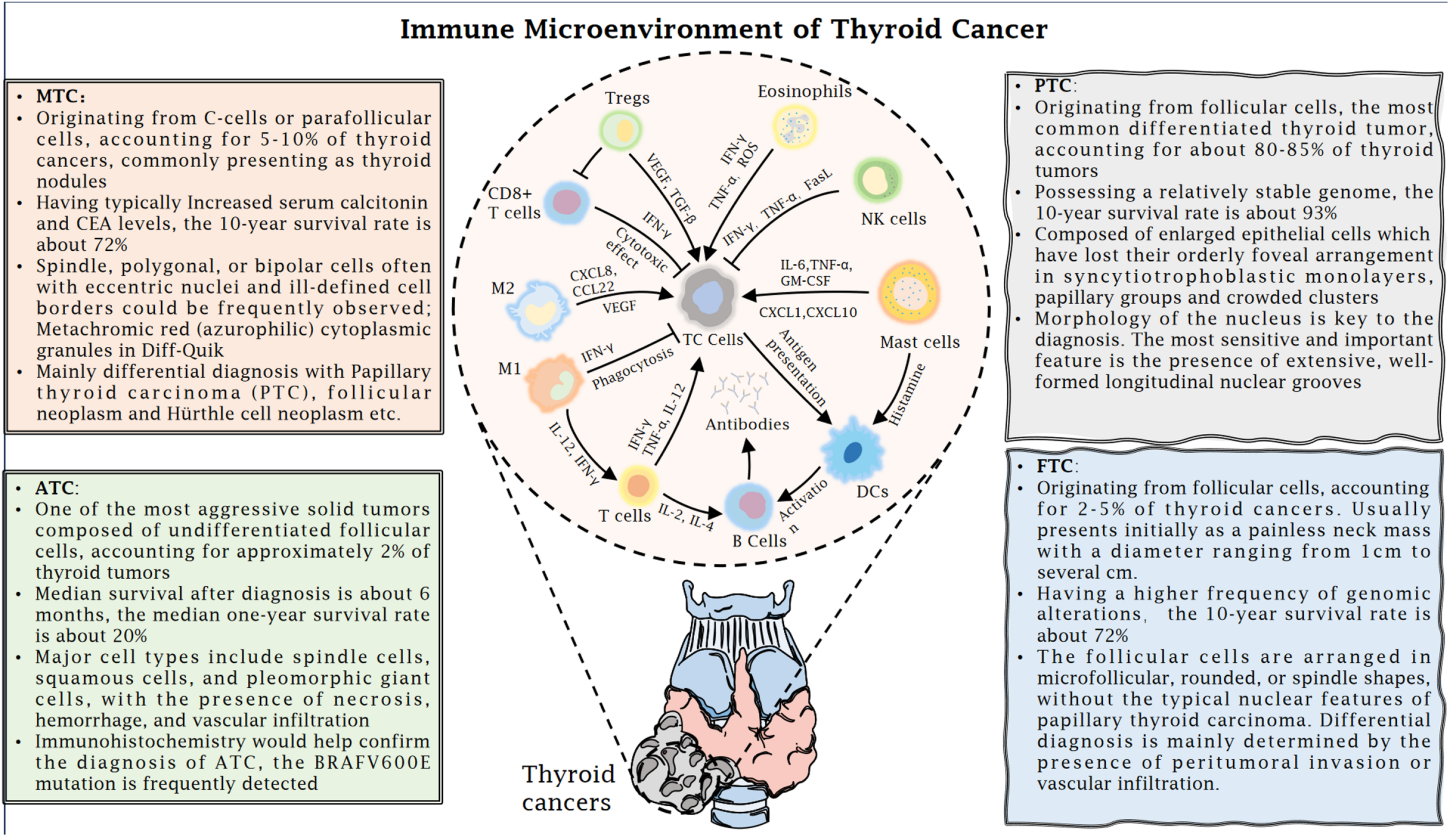
Mechanisms of ferroptosis. Fe3^+^ bound to transferrin enters the cell via the transferrin receptor and is then reduced to Fe2^+^ by STEAP3 in the lysosome. Fe2^+^ could enter the cytoplasm through DGT1, together with Fe2^+^ from degraded heme and Fe2^+^ released by NCOA4‐mediated ferritinophagy to form LIP. Extracellular Fe2^+^ could also penetrate directly into the cell via DGT1 located on the membrane and participate in the formation of LIP. Fe2^+^ within the LIP has three main endings: Passing out of the cell via ferroportin, being stored by binding to ferritin, and generating ROS by Fenton reaction. ROS generated by the Fenton reaction along with other metabolic processes could facilitate the initiation of lipid peroxidation, and ultimately, cause ferroptosis together with ALOXs. System Xc‐ and GPX4 are the main components that inhibit lipid peroxidation, while AIFM2‐CoQ10, GCH‐1‐BH4 and DHODH‐CoQH2 also prevent the occurrence of lipid peroxidation in the cell.

Ordinarily, several mechanisms allow cells to prevent peroxidation reactions from occurring. System Xc‐ and glutathione peroxidase 4 (GPX4) are two major components of the antioxidant process, and their inactivation is directly involved in the initiation of ferroptosis.^60^, ^61^ System Xc‐Transporter proteins could carry glutamate out of the cell and transport cystine into the cell simultaneously.^62^ Cystine could be reduced to cysteine, which then participates in the biosynthesis of reduced glutathione (GSH). GPX4 can utilise GSH to reduce PUFAs‐OOH to PUFAs‐OH, thereby preventing the accumulation of LPO and the inhibition of ferroptosis.^63^, ^64^ Therefore, the repression of system Xc‐ could reduce GSH production by decreasing cystine input, thus inhibiting the function of GPX4 and resulting in the accumulation of LPO.^65^, ^66^ Several other factors, such as the apoptosis‐inducing factor alpha‐related 2 (AIFM2)‐Coenzyme Q10 (CoQ 10) pathway, the GTP cyclohydrolase‐1 (GCH1)‐tetrahydrobiopterin (BH4) pathway, the dihydroorotate dehydrogenase (DHODH)‐CoQH2 pathway and some tumour suppressors are also involved in the regulation of ferroptosis^67^, ^68^, ^69^ (Figure 2).

## 3 THE ROLE AND MECHANISMS OF FERROPTOSIS IN REMODELLING TC PROGRESSION

### 3.1 M6A in ferroptosis regulation

RNA methylation is a chemical modification phenomenon in which the methyl adenine of RNA is selectively modified by the addition of a methyl group, catalysed by methyltransferases, with the main form being m6A methylation.^70^, ^71^, ^72^ The m6A modification is one of the widely known chemical modifications of mammalian RNAs and occurs in different types of RNA molecules such as mRNAs, rRNAs, miRNAs and lncRNAs.^73^, ^74^ Epigenetic regulation mediated by m6A modifications performs essential roles in regulating post‐transcriptional processing, stability, splicing, degradation, translation and function of RNAs and profoundly affects the expression of a diversity of physiologically and pathologically relevant genes.^75^ The m6A methylation modification is a dynamic and reversible process that is synergistically regulated by three enzymes with different functions, including methyltransferases (writers), demethylases (erasers) and binding proteins (readers).^76^, ^77^, ^78^

ALKBH5 is a tumour suppressor that is lowly expressed in some tumours and can behave as a tumour suppressor in an m6A‐dependent manner.^79^, ^80^ ALKBH5 expression showed a trend of decreased expression in TC tissues and cells.^81^ In biological behavioural investigations, the overexpression of ALKBH5 increased Fe2^+^ and ROS content, down‐regulated the expression of GPX4 and SLC7A11 and consequently restricted TC cell proliferation. Furthermore, both in vivo and ex vivo studies confirmed that ALKBH5 inhibited TC progression by reducing the m6A level of TIAM1, confirming the core mechanism of ferroptosis triggered by the ALKBH5‐TIAM1‐Nrf2/HO‐1 axis.^81^ Therefore, ALKBH5 is expected to be a potential targeted target for TC diagnosis and treatment.

Fat mass and obesity‐associated protein (FTO) is the first identified m6A demethylase, and its imbalanced expression is critical in the development and progression of several tumours, including TC, renal cancer, breast cancer and bladder cancer.^82^, ^83^ Interventional therapies targeting FTO can inhibit tumour growth, enhance immune effects, inhibit drug resistance and sensitise chemotherapeutic agents through multiple biological effects. FTO expression was significantly reduced in PTC tissues.^84^ From a cell biological perspective, FTO was able to inhibit PTC independently by down‐regulating SLC7A11 in m6A.^84^ FTO might therefore act as an oncogene for PTC, providing a novel m6A‐ferroptosis‐based strategy for the treatment of PTC.

### 3.2 NcRNA in ferroptosis regulation

Non‐coding RNAs (ncRNAs) can regulate the expression of multiple downstream gene targets and related pathways, and the most popular and intensively investigated are mainly microRNAs (miRNAs), long non‐coding RNAs (ncRNAs) and circular RNAs (circRNAs).^85^, ^86^, ^87^ NcRNAs are involved in almost all aspects of tumours, including pathogenesis, diagnosis, treatment and prognosis evaluation.^88^ NcRNAs have important roles in mediating cellular ferroptosis. NcRNAs associated with ferroptosis are key molecules affecting TC progression and may serve as effective targets for TC diagnosis and treatment.

LncRNAs are RNAs greater than 200 nt in length transcribed by RNA polymerase with conserved secondary structures, most of which do not encode proteins.^89^, ^90^ Studies have shown that lncRNAs are implicated in a multitude of biological processes, including reprogramming of pluripotent stem cells, oncogenic progression and cell cycle regulation. lncRNAs can regulate a diversity of functions such as chromatin structure and function, transcription, RNA splicing, stability and translation as well as cecRNA regulation through interactions with DNA, RNA and proteins.^91^, ^92^ Huang et al.^93^ showed that lncRNA CERS6‐AS1 and LASP1 are indicators of high expression in TC tissues and cells and were closely associated with poor prognosis. The expression level and prognosis of miR‐497‐5p were opposite to that of lncRNA CERS6‐AS1. Mechanistically, inhibition of CERS6‐AS1 could decrease the cell survival of PTC and enhance the ferroptosis response by regulating the miR‐497‐5p/LASP1 axis.

CircRNAs are single‐stranded, covalently enclosed RNA molecules that are abundantly present in the eukaryotic transcriptome.^94^ CircRNAs can perform biological functions as transcriptional regulators, microRNA sponges and protein scaffolds. Most of the circRNAs are composed of exonic sequences, which are conserved in diverse species, with tissue and developmental stage‐specific expression.^95^, ^96^ CircKIF4A is highly expressed in tumour tissues such as lung cancer, glioma, triple‐negative breast cancer (TNBC), ovarian cancer and TC.^97^, ^98^, ^99^ Chen et al.^100^ demonstrated that circKIF4A regulated the miR‐1231‐GPX4 axis by competing endogenous RNA mechanisms and caused PTC cell growth and migration, facilitating PTC malignant progression and suppressing ferroptosis effects. In another study, circ_0067934 upregulated the expression of SLC7A11 and promoted the proliferation of TC cells by sponge‐binding miR‐545‐3p.^101^ Thus, inhibition of the circ_0067934/miR‐545‐3p/SLC7A11 axis was able to arrest TC progression by enhancing TC ferroptosis.

### 3.3 GPX4 in ferroptosis regulation

GPX4 has received great attention for its unique ability to resist membrane lipid oxidation. GPX4 is a central modulator of ferroptosis, and direct targeting of GPX4 to induce ferroptosis in tumour cells is a prospective strategy for the exploitation of therapeutic anticancer drugs.^102^ Knockdown of GPX4 inhibited the proliferation of TC cells and induced ferroptosis. Thus, GPX4 is involved in the evolution of TC.^103^ Inhibition of GPX4 by drugs such as RSL3 and genetic interventions could induce ferroptosis, mTOR pathway suppression, and DNA damage repair response.^104^ Notably, one study explored the mechanisms associated with GPX4 inhibitors on TC cells and 3‐D spheroid in vitro models.^105^ The results suggested that GPX4 inhibitors induced ferroptosis response, ROS elevation, GSH depletion, as well as inhibited tumour cell migration, increased DNA damage and inhibited the mTOR pathway. Ultimately, GPX4 inhibitors have different effects on TC cells with different mutational profiles, such as BRAF, RAS, TERT promoter and PIK3CA co‐mutant PTC cells and on both monolayer TC cells and 3‐D PTC spheroids.

### 3.4 Other key proteins participating in ferroptosis regulation

E26 transformation‐specific (ETS) variant 4 (ETV4), an ETS family transcription factor, has been demonstrated to be an accomplice in the metastasis and progression of many tumours and a key factor in patient survival.^106^ ETV4 regulates chemokine expression to recruit tumour‐associated lymphocytes such as neutrophils, for promoting tumour metastasis and organ colonization.^107^ Targeting ETV4 may be an effective strategy to inhibit tumour metastasis.^108^, ^109^ Wang et al.^110^ demonstrated that ETV4, a transcription factor upregulated in PTC tissues and cells, promoted PTC cell proliferation. Knockdown of ETV4, meanwhile, inhibited PTC progression by down‐regulating the expression of SLC7A11, which promoted the tumour ferroptosis effect.

CD71, also known as transferrin receptor 1 (TfR1), is a cell‐surface proliferation marker involved in cellular iron uptake.^111^ D'Aprile et al.^112^ showed that the tissue expression levels of CD71 were higher on ATC than on FTC. Subsequently, cellular experiments using FTC‐133 (follicular) and 8505C (anaplastic) confirmed that 8505C could tolerate ferroptosis better than FTC‐133. In this process, 8505C might function in resisting ferroptosis and preserving cell viability by regulating CD71, which engaged in iron internalization. This evidence suggested that ATC cells could increase iron overload tolerance by decreasing CD71 levels.

**FIGURE 3:**
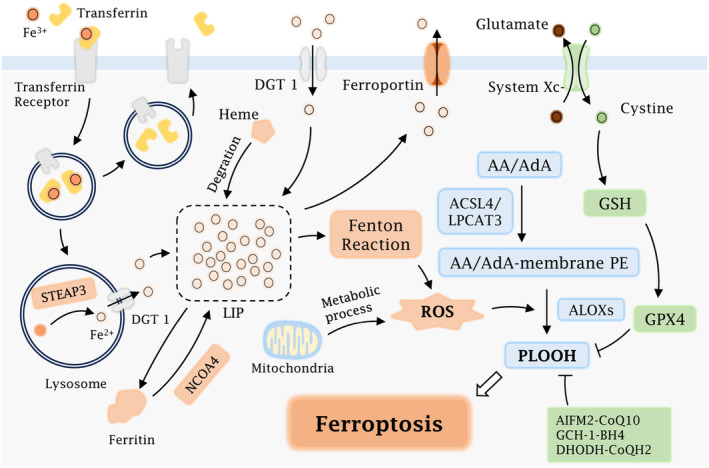
The role and mechanisms of ferroptosis in remodelling TC progression. M6A modification: ALKBH5 could downregulate the m6A level of TIAM1, thereby promoting ferroptosis through the TIAM1/Nrf‐2/HO‐1 axis. FTO promoted the downregulation of m6A in SLC7A11 and inhibited SLC7A11 function, facilitating ferroptosis in TC cells. NcRNAs regulation: The lncRNA CERS6‐AS1 could inhibit ferroptosis via the miR‐417‐5p/LASP1 axis, while circ_KIF4A suppressed ferroptosis via miR‐1231/GPX4 axis. Circ_0067934 could enhance the expression of SLC7A11 via sponge‐binding with miR‐545‐3p, thus preventing ferroptosis and promoting the proliferation of TC cells. GPX4: GPX4 is an essential factor in preventing ferroptosis, which could reduce intracellular ROS levels, lipid peroxidation products, and DNA damage, resulting in promoted TC cell proliferation and migration. ETV4: ETV4 regulates the expression of chemokines, and it also inhibits ferroptosis in tumour cells via SLC7A11. TFR1: TFR1 is a pivotal component of Fe intake, and ATC cells could enhance their resistance to ferroptosis by down‐regulating TFR1. SIRT6: USP10 could suppress erastin‐induced ferroptosis by SIRT6‐induced GPX4 promotion. Meanwhile, SIRT6 could in turn enhance NCOA4‐mediated ferritinophagy, increasing the levels of LIP and enhancing ferroptosis.

Sirtuin 6 (SIRT6) is a key member of the mammalian sirtuin family of conserved nicotinamide adenine dinucleotide (NAD^+^)‐dependent deacetylases, essentially a chromatin deacetylase.^113^ SIRT6 is responsible for regulating metabolism, DNA damage repair and ageing and is participating in the regulation of numerous diseases such as cancer, inflammation, diabetes and cardiovascular disease.^114^ The role of SIRT6 in tumours is double‐edged sword‐like, exerting promotional and inhibitory effects in different tumour types.^115^, ^116^ Lian et al.^117^ reported that ubiquitin‐specific peptidase 10 (USP10) is a highly expressed marker of oncogenic activity, which is closely related to the prognosis of TC. USP10 not only increased TC cell proliferation and motility but also attenuated the phenomenon of erastin‐induced ferroptosis, which was accompanied by reduced iron, malondialdehyde and lipid ROS levels. This process was dependent on the finding that USP10 could facilitate the expression of GPX4 by increasing SIRT6, which in turn attenuated ferroptosis. In addition, SIRT6 was capable of triggering NCOA4‐dependent ferritinophagy, thereby heightening sensitivity to ferroptosis.^118^ In an in vivo model, ferroptosis inducer sulfasalazine effectively inhibited SIRT6‐upregulated TC, reflecting the effectiveness of TC therapy targeting SIRT6 and ferroptosis (Figure 3).

## 4 FERROPTOSIS‐RELATED APPLICATIONS IN TC DIAGNOSIS

Currently, the overall diagnostic and therapeutic outcome of TC is relatively definitive and immediate. The diagnosis and prognosis evaluation of TC mainly relies on imaging, pathology and serologic examination.^119^, ^120^ The gold standard for the diagnosis of TC is the result of pathological examination, which can be definitively diagnosed through cell morphology, histopathological grading, thickness of thyroid peritoneum, thyroid function measurement and other examinations.^121^, ^122^ Many studies now use bioinformatics methods to screen for associations between the expression abundance of genes and tumour prognosis, using publicly available or self‐sequenced databases.^123^, ^124^, ^125^ These validated single indicators, or a combination of indicators for the construction of predictive signatures, can not only assess the prognosis of patients but also potentially provide valuable tools and strategies for the characterisation of the immune microenvironment, the evaluation and guidance of drug resistance and the monitoring of disease therapies.^120^, ^126^ A number of relevant indicators and risk modelling studies are also gradually unfolding in the TC applications.

GPX4 may be a key factor in the survival impact of TC and is a valuable indicator that can predict the prognosis of TC. GPX4 is highly expressed in TC tissues compared to normal tissues, associated with T3–T4 stages and pathological stages III–IV and closely related to biological activities such as muscle contraction, contractile fibres, complement and coagulation cascade.^103^ Fanet et al. screened BID and CDKN2A for prognostic modelling, which could effectively predict the prognosis of TC patients and guide clinical treatment, and found that BID and CDKN2A were prognostic risk factors and protective factors, respectively.^127^ Ferroptosis and immune might be engaged in the advancement of TC through the antitumor immunity and BRAF/NRAS/HRAS mutation.

The construction of risk models is dependent on the processes of key gene mining, modelling and evaluation.^128^, ^129^ Wang et al.^130^ utilised the Gene Expression Omnibus (GEO) and The Cancer Genome Atlas (TCGA) databases and screened FRGs, and accordingly established a relevant prognostic model based on AKR1C3, BID, FBXW7, GPX4 and MAP3K5. This predictive model was effective in evaluating the three‐year survival rate of TC patients. Qin et al.^131^ also screened FRGs and the corresponding lncRNAs using TCGA and FerrDb databases and constructed a novel prognostic model FRLs. This model was considered as a TC‐independent prognostic indicator, which showed that the prognosis of patients in the high‐risk group was worse than that of patients in the low‐risk group and that the activation of anticancer immune‐related pathways as well as the activation of immune cells such as plasma cells and immune checkpoint molecules such as CTLA‐4 and LAG3 were significantly increased in the low‐risk group. Another study also constructed a predictive model based on 8 FRGs, including DPP4, TYRO3, TIMP1, CDKN2A, SNCA, NR4A1, IL‐6 and FABP4.^127^ The modelled score was a potentially independent predictor of clinical diagnosis and prognosis that could indicate differences in immune cell infiltration.

Generally speaking, the prognosis of PTC patients is satisfactory, and its prognosis is related to the size of the tumour, the number of lymph node metastases and the invasion of distant tissues.^132^ The construction of some risk models is conducive to a more abundant presentation of the tumour environment, prognosis and treatment information of PTC. Huang et al.^133^ constructed a PTC prediction model containing three FRGs, including HSPA5, AURKA and TSC22D3. Patients in the high‐risk group had a shorter survival. Functional analysis showed that set enrichment analysis (ssGSEA), immune cell infiltration, tumour immune microenvironment (TME), human leukocyte antigen (HLA), and tumour mutational burden (TMB) were strongly linked to ferroptosis mutations. Yang et al.^134^ constructed a prognostic model based on eight FRGs, including DPP4, GPX4, GSS, ISCU, MIOX, PGD, TF and TFRC. The high‐ and low‐risk groups obtained according to the scores could significantly reflect the high and low OS rates of patients with PTC and the immune cell types and their expression of the significant differences. Similarly, Shi et al.,^135^ Qian et al.^136^ and Lin et al.^137^ constructed a FRG model that was successfully used for prognostic prediction of PTC. All of these studies suggest that signatures based on FRG can predict the prognosis of patients with PTC.

Both lncRNA and ferroptosis are undoubtedly key regulators in TC progression.^138^, ^139^ Then, model construction based on ferroptosis‐associated lncRNA may be effective in providing disease prognostic information and correlating therapeutic strategies. For example, Lin et al.^140^ successfully constructed a ferroptosis‐associated lncRNA prognostic model (Ferr‐LPM), in which higher scores implied shorter overall survival (OS). In addition, the Ferr‐LPM was able to have key features indicative of and modulating TME, and the Ferr‐LPM score was inversely correlated with tumour purity and positively correlated with immune checkpoint blockade (ICB) response. Ferr‐LPM score correlated inversely with tumour purity and positively with ICB response. Therefore, Ferr‐LPM is a highly efficient tool for TC diagnosis and immune evaluation.

## 5 FERROPTOSIS‐TARGETED DRUGS IN TC THERAPY

In terms of treatment, ferroptosis is strongly associated with tumour resistance, treatment sensitization, radiotherapy and immunotherapy resistance.^141^ Accordingly, therapeutic strategies based on the key targets of ferroptosis, such as GPX4, ferroptosis suppressor protein 1 (FSP1) and GCH1, are capable of reversing cancer drug resistance and interrupting tumour progression.^142^ Relatively speaking, ferroptosis‐targeted therapeutic strategies have been widely reported in other more malignant tumour types, while relatively few studies have been conducted in TC, probably due to the relatively good overall prognosis of TC, which has received less research attention. Some of the small molecule inhibitors associated with ferroptosis in TC include anlotinib, neferine, vitamin C and a diaryl ether derivative, most of which are competent to induce tumour cell death by inducing ferroptosis in tumour cells and may amplify the immune effect.

Anlotinib is a small‐molecule inhibitor of the multi‐target tyrosine kinase receptor and can effectively curb tumour growth and angiogenesis.^143^, ^144^ Wu et al.^145^ demonstrated that anlotinib could effectively inhibit the activity of some human ATC cell lines, including KHM‐5 M, C643 and 8505C. In mechanistic explorations, anlotinib significantly reduced ferroptosis‐associated markers, including transferrin, ferritin light chain (FTL) and GPX4, but not pyroptosis, apoptosis and necroptosis. Moreover, protective autophagy was initiated under anlotinib stimulation, and autophagy interruption potentiated anlotinib‐mediated ferroptosis and tumour‐killing responses in vivo and in vitro. This study fully substantiated the potential role of autophagy‐ferroptosis in the inhibition of tumour and angiogenesis in ATC by amlotinib, and may therefore contribute to the development of novel therapeutic strategies for ATC treatment.

Neferine is a bisbenzylisoquinoline alkaloid isolated from lotus seed, possessing a wide range of pharmacological effects such as antihypertensive, antiarrhythmic, anti‐inflammatory, anticancer and antioxidant.^146^, ^147^ Neferine enhanced the apoptosis of IHH‐4 and CAL‐62 cells and potentiated the ferroptosis effect of these cells.^148^ Further animal experiments showed that neferine could effectively promote the ferroptosis effect in tumours and consequently kill TC cells by inhibiting the Nrf2/HO‐1/NQO1 pathway. Vitamin C, an ascorbic acid vitamin, is recognised for its ability to regulate immune function, act as an epigenetic modulator, regulate programmed cell death and tumoricidal properties.^149^ Vitamin C significantly inhibited the growth of ATC cells by inducing free iron release, ROS generation and sustained lipid peroxidation, which ultimately led to the ferroptosis effect of ATC.^150^

BRAFV600E is a frequent mutation type in PTC, and its relevant inhibitors have been widely developed and applied, but the associated drug resistance is a growing challenge.^151^, ^152^ Pamarthy et al.^153^ developed a Diaryl ether derivative capable of targeting and inhibiting the expression level of GPX4, decreasing mitochondrial polarization and effectively inducing the ferroptosis effect in TC cells by reducing mitochondrial polarization and effectively inducing ferroptosis. This compound was recognized by the authors as a promising ferroptosis‐inducing compound for TC therapy.

## 6 DISCUSSION

The mechanisms of TC progression, metastasis and recurrence remain a point of interest in the current field of tumour therapy.^154^, ^155^ PCD‐related death modalities, especially ferroptosis, are closely connected with multiple biological behaviours of TC. Here, we focus on elucidating the functional, mechanistic, diagnostic and therapeutic aspects of ferroptosis in TC. Nevertheless, there are still many urgent issues that need to be tackled in this area.

Mechanistically, ferroptosis is currently far less explored in TC than in other malignant tumours. Many cancer‐related genes and signalling pathways can regulate ferroptosis, and some signalling molecules generated by ferroptosis can also be the source of activation of other pathways, which in turn constitutes a complex positive or negative feedback network. NcRNAs are also gradually recognised as key mediators in the regulation of ferroptosis.^156^ NcRNAs are still mainly regulated in TC through the ceRNA mechanism to regulate gene expression and reshape tumour behaviour. However, the known quantity of miRNAs, lncRNAs and circRNAs in TC is still relatively small, and all of them also basically remain in sample validation and cell and animal exploration. In addition, multiple forms of PCD exist in TC, including apoptosis, pyroptosis, necroptosis and autophagy.^157^, ^158^, ^159^ There exists a close interplay mode between these modes of death, especially autophagy and ferroptosis, which can profoundly and complexly affect the course of TC. Compared to other forms of PCD, the study of ferroptosis in TC is still in its early stages. How to decipher this complex interaction needs to be studied in depth.

Ferroptosis significantly interacts with the immune system, influencing the progression and treatment of various cancer types, including TC. The release of damage‐associated molecular patterns (DAMPs) during ferroptosis can stimulate immune to response, thus attracting immune cells to the tumour microenvironment.^160^ For instance, ferroptotic cells can release high‐mobility group box 1 (HMGB1), which activates DCs and promotes the presentation of tumour antigens to T cells, thereby enhancing anti‐tumour immunity.^161^ Moreover, the ferroptosis‐induced release of lipid peroxidation products can modulate the activity of macrophages and NK cells, further influencing the immune landscape of TC.^162^ Macrophages can shift into different phenotypes based on the stimulated signals from the tumour microenvironment, and ferroptosis can promote the polarization of macrophages towards an M1 phenotype, associated with pro‐inflammatory and anti‐tumour activities.^163^ NK cells, on the other hand, can recognise and kill ferroptotic cells, adding another layer of immune surveillance. The interplay between ferroptosis and the immune system also has implications for immunotherapy. Immune checkpoint inhibitors, such as anti‐PD‐1 and anti‐CTLA‐4 antibodies, can be combined with ferroptosis inducers to enhance therapeutic efficacy.^164^, ^165^ By promoting ferroptosis in tumour cells, these combination therapies can increase the immunogenicity of the tumour, making it more susceptible to immune‐mediated destruction. Despite its potential, the association between ferroptosis and immune cells like CD8+ T cells, macrophages, NK cells and DC cells in TC has not received enough attention. Improving the specificity of ferroptosis inducers and strictly controlling their dosage is essential to enhance the immunocidal effect and reduce adverse effects on normal tissues. Understanding these interactions can provide new insights into developing combination therapies that harness both ferroptosis and the immune response, potentially improving treatment outcomes for TC patients.

In terms of diagnostics, FRGs are still in the initial stages of cellular and sample validation, which is not yet sufficient for diagnostic practice in TC clinics. Although these models are economical and have abundant predictive information, validation of external cohorts and real‐world data is still relatively scarce. The validity of prognostic models is difficult to confirm in real clinical diagnosis. In addition, the spatial expression pattern, abundance and detailed molecular mechanism function of FRGs at the protein level have not been explored in corresponding studies.^166^ These models are not similar to conventional methods. First of all, because these are risk models constructed from risk genes, they differ from conventional measurement parameters in terms of metrics. Second, these models respond to richer information about the diagnosis and treatment of the disease, a feature that is not matched by conventional methods. The most important thing is that these TC prediction models based on FRGs are not a replacement of the conventional classical methods but serve as a reference to become a beneficial supplement.

The unique characteristics of ferroptosis, including its iron dependence and lipid peroxidation damage, distinguish it from other forms of PCD, highlighting its potential in cancer therapy. The therapeutic effects of ferroptosis in various tumours are becoming increasingly prominent, but relevant studies in TC are not sufficiently advanced. By promoting the Fenton reaction, inhibiting the Xc‐system/GPX4 and regulating the level of lipid peroxidation in tumour cells, these can effectively activate and amplify the iron death effect and kill tumours. Although ferroptosis inducers have shown promising anti‐tumour effects in vitro and in animal models, their clear therapeutic effects in clinical applications are yet to be confirmed. Preliminary studies have demonstrated that ferroptosis inducers can significantly inhibit TC cell proliferation and induce cell death, but these results have not yet been translated into clinical treatment protocols. First, targeting ferroptosis and inducing tumour cell ferroptosis can certainly produce certain anti‐tumour effects, and ferroptosis‐targeted as well as nanotherapies and combination therapies developed based on this have therapeutic strategies to amplify ferroptosis.^167^ Multifaceted combination therapies that synergize the ferroptosis of tumour cells, including immunotherapy, combined with other PCD therapies, gene therapy, radiotherapy and other modalities, will become a future therapeutic trend. In addition, the ferroptosis‐related nano‐delivery platform can excellently integrate different therapeutic agents for synergistic therapy, which not only prolongs the onset of ferroptosis in cells but also improves the targeting of therapeutic tumours.^168^ Therefore, ferroptosis‐related targeted therapy, integrative therapy and nanotherapeutics have great potential in cancer therapy, hence providing diverse options and strategies for the treatment of malignant TCs.

In conclusion, ferroptosis is involved in various aspects of TC initiation, malignant progression, diagnosis and treatment by regulating the malignant biological behaviour of tumour cells through diverse mechanisms. Specifically, epigenetic mechanisms represented by m6A, NcRNA and some key regulatory proteins, such as GPX4, CD71, ETV4 and SIRT6, interfere with TC progression by regulating tumour cell ferroptosis. Based on ferroptosis‐related mRNAs and lncRNAs can be used to construct TC‐related risk prediction models. Finally, therapeutic approaches targeting ferroptosis can provide novel curative perspectives for combating and management of TC.

## AUTHOR CONTRIBUTIONS

**Lin Yin:** Conceptualization (equal); data curation (equal); formal analysis (equal); funding acquisition (equal); investigation (equal); methodology (equal); project administration (equal); resources (equal); software (equal); supervision (equal); validation (equal); visualization (equal); writing – original draft (equal); writing – review and editing (equal). **Xiaodan Luo:** Conceptualization (equal); data curation (equal); investigation (equal); methodology (equal); project administration (equal); resources (equal); validation (equal). **Xian Zhang:** Conceptualization (equal); data curation (equal); formal analysis (equal); funding acquisition (equal); investigation (equal); methodology (equal); project administration (equal); resources (equal); software (equal); supervision (equal); validation (equal); visualization (equal); writing – original draft (equal); writing – review and editing (equal). **Bomin Cheng:** Conceptualization (equal); data curation (equal); formal analysis (equal); funding acquisition (equal); investigation (equal); methodology (equal); project administration (equal); resources (equal); software (equal); supervision (equal); validation (equal); visualization (equal); writing – original draft (equal); writing – review and editing (equal).

## CONFLICT OF INTEREST STATEMENT

The authors declare that this study does not involve any business or financial relationship that could be considered a potential conflict of interest.

## Data Availability

The data that support the findings of this study are available on request from the corresponding author. The data are not publicly available due to privacy or ethical restrictions.
